# Neuropeptide complexity in the crustacean central olfactory pathway: immunolocalization of A-type allatostatins and RFamide-like peptides in the brain of a terrestrial hermit crab

**DOI:** 10.1186/1756-6606-5-29

**Published:** 2012-09-11

**Authors:** Marta A Polanska, Oksana Tuchina, Hans Agricola, Bill S Hansson, Steffen Harzsch

**Affiliations:** 1 Department of Animal Physiology, Zoological Institute, Faculty of Biology, University of Warsaw, 1 Miecznikowa Street, 02-096, Warsaw, Poland; 2 Department of Evolutionary Neuroethology, Max Planck Institute for Chemical Ecology, Beutenberg Campus, Hans-Knöll-Str. 8, D-07745, Jena, Germany; 3 Zentrum für Molekulare Biomedizin, Friedrich-Schiller-Universität Jena, Philosophenweg 12, D-07743, Jena, Germany; 4 Zoological Institute and Museum, Cytology and Evolutionary Biology, Ernst Moritz Arndt University, Greifswald, 17497, Germany

**Keywords:** *Coenobita clypeatus*, Hermit crab, Olfactory lobe, Central complex, Neuropeptide, Immunhistochemistry, Allatostatin

## Abstract

**Background:**

In the olfactory system of malacostracan crustaceans, axonal input from olfactory receptor neurons associated with aesthetascs on the animal’s first pair of antennae target primary processing centers in the median brain, the olfactory lobes. The olfactory lobes are divided into cone-shaped synaptic areas, the olfactory glomeruli where afferents interact with local olfactory interneurons and olfactory projection neurons. The local olfactory interneurons display a large diversity of neurotransmitter phenotypes including biogenic amines and neuropeptides. Furthermore, the malacostracan olfactory glomeruli are regionalized into cap, subcap, and base regions and these compartments are defined by the projection patterns of the afferent olfactory receptor neurons, the local olfactory interneurons, and the olfactory projection neurons. We wanted to know how neurons expressing A-type allatostatins (A-ASTs; synonym dip-allatostatins) integrate into this system, a large family of neuropeptides that share the C-terminal motif –Y*X*FGLamide.

**Results:**

We used an antiserum that was raised against the A-type *Diploptera punctata* (Dip)-allatostatin I to analyse the distribution of this peptide in the brain of a terrestrial hermit crab, *Coenobita clypeatus* (Anomura, Coenobitidae). Allatostatin A-like immunoreactivity (ASTir) was widely distributed in the animal’s brain, including the visual system, central complex and olfactory system. We focussed our analysis on the central olfactory pathway in which ASTir was abundant in the primary processing centers, the olfactory lobes, and also in the secondary centers, the hemiellipsoid bodies. In the olfactory lobes, we further explored the spatial relationship of olfactory interneurons with ASTir to interneurons that synthesize RFamide-like peptides. We found that these two peptides are present in distinct populations of local olfactory interneurons and that their synaptic fields within the olfactory glomeruli are also mostly distinct.

**Conclusions:**

We discuss our findings against the background of the known neurotransmitter complexity in the crustacean olfactory pathway and summarize what is now about the neuronal connectivity in the olfactory glomeruli. A-type allatostatins, in addition to their localization in protocerebral brain areas, seem to be involved in modulating the olfactory signal at the level of the deutocerebrum. They contribute to the complex local circuits within the crustacean olfactory glomeruli the connectivity within which as yet is completely unclear. Because the glomeruli of *C. clypeatus* display a distinct pattern of regionalization, their olfactory systems form an ideal model to explore the functional relevance of glomerular compartments and diversity of local olfactory interneurons for olfactory processing in crustaceans.

## Background

Neurochemicals, e.g. biogenic amines and neuropeptides, play an important role in controlling and modulating nervous function in all organisms. Much attention is currently being focused on understanding the neuropeptide complexity of the arthropod nervous system [1-3]. In crustaceans, more than 30 major neuropeptide families are known [4-6]. The olfactory lobes in the crustacean brain are primary processing centers where chemosensory input from first pair of antennae is relayed on local olfactory interneurons and projection neurons [7-9]. The olfactory interneurons are involved in modulation of olfactory processing and synthesize a vast variety of different neurotransmitters including serotonin, histamine and GABA as well as many different neuropeptides such as RFamide related peptides, substance P, small cardiactive peptide b, orcokinins, SIFamide, and tachykinin-related peptides [10-18], review [19] In the present contribution we are interested in the distribution of the allatostatins in the brain, specifically the central olfactory pathway, of a decapod crustacean, the hermit crab *Coenobita clypeatus* (Anomura, Coenobitidae) because these peptides previously had been shown to be abundant within the insect olfactory pathway [1].

The A-type allatostatins (A-ASTs; synonym dip-allatostatins) constitute a large family of neuropeptides that were first identified from the cockroach *Diploptera punctata* and that share the C-terminal motif –Y*X*FGLamide [reviews [1,20,21]. In decapod crustaceans, almost 20 native A-ASTs and related peptides were initially identified from extracts of the thoracic ganglia of the shore (green) crab *Carcinus maenas*[22], and shortly after several other A-ASTs were isolated from the freshwater crayfish *Orconectes limosus*[23]. Meanwhile, the family of crustacean A-ASTs has substantially grown to several dozens of representatives [6] with additional members being discovered in the prawns *Penaeus monodon*[24] and *Macrobrachium rosenbergii*[25], in the brachyuran crabs *Cancer borealis*[26] and *Cancer productus*[27], the crayfish *Procambarus clarkii*[17], the lobster *Homarus americanus*[4,28,29], the shrimps *Litopenaeus vannamei*[5] as well as a non-malacostracan crustacean, the copepod *Calanus finmarchicus*[30].

Initial immunolocalization studies showed that A-ASTs are present in the stomatogastric nervous system and pericardial organs of crayfish, lobsters and brachyuran crabs [23,31,32] and also in the neuroendocrine organs of the water flea *Daphnia pulex*[33]. Skiebe [31,32] suggested A-ASTs in crustaceans to function as circulating hormones and modulators that are released by interneurons, by sensory neurons and possibly also by motoneurons. Physiological experiments in decapods crustaceans have shown that A-ASTs exert an inhibitory modulatory effect on the pyloric and gastric rhythms generated by the stomatogastric ganglion [23,31,34] and increase spike-time precision of sensory neurons associated with the stomatogastric nervous system [35]. These peptides were also shown to inhibit contractions of the crayfish hindgut [23]. At cholinergic and glutaminergic neuromuscular junctions, A-ASTs reduce the amplitude of excitatory junctional potentials and excitatory junctional currents [34] and decrease neuromuscular synaptic transmission by other pre- and postsynaptic mechanisms [36]. The peptides also decrease the cycle frequency of the cardiac ganglion by modulating spike frequency and number of cardiac motoneurons [37]. In summary, ASTs in malacostracan crustaceans have been documented to exert an inhibitory neuro-/myomodulatory function [6].

Concerning the central nervous system of malacostracan crustaceans, immunolocalization experiments have indicated the presence of interneurons that express A-ASTs in the eyestalk ganglia [23,38,39] and the median part of the brain of various crayfish, a prawn and anomuran and brachyuran crabs [17,23,39-41]. They are also present in the nervous system of a non-malacostracan crustacean, the copepod *Calanus finmarchicus*[42]. In the current study we set out to analyze the role of A-ASTs in the crustacean brain in more detail by analyzing their distribution in the terrestrial hermit crab *Coenobita clypeatus* (Anomura, Coenobitidae) focussing on its central olfactory pathway. The genus *Coenobita* includes 15 species of shell-carrying land hermit crabs that display a fully terrestrial life style and are dependent on the ocean only to release their larvae [43]. We recently explored the general brain anatomy of *C. clypeatus* with regard to possible adaptations of the nervous system to their terrestrial life style [18,44]. In the present report, beyond general neuroanatomy, we wanted to gain deeper insights into the arrangement of specific classes of neurons. To that end, we used an antiserum that was raised against the A-type *Diploptera punctata* (Dip)-allatostatin I, APSGAQRLYGFGLamide [45] and that previously has been used to localize A-ASTs in crustacean and insect nervous systems [23,32,41,45,46]. We supplement these data with double labeling experiments with an antiserum against RFamide-related peptides which previously were shown to be localized within this animal’s olfactory system [18]. We compare our immunohistochemical results to the distribution pattern of A-ASTs in the insect olfactory pathway.

## Results

### Overview of the *Coenobita clypeatus* brain and A-type allatostatin-like immunoreactivity (ASTir)

**Figure 1:**
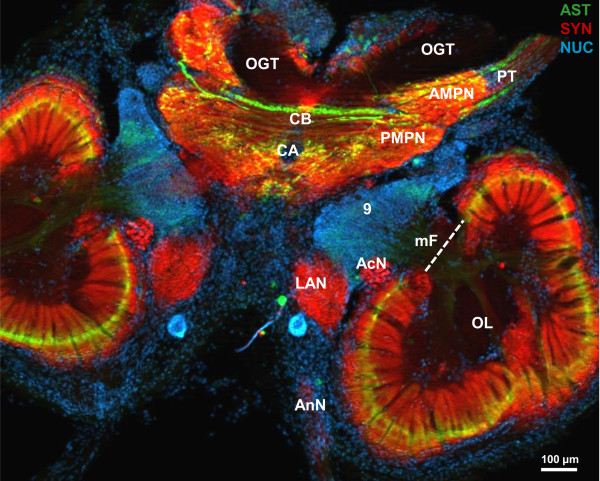
**Overview of the *Coenobita clypeatus* median brain (horizontal section, conventional fluorescence with Apotome structured illumination) triple labelled for A-type allatostatin-like immunoreactivity (ASTir; green), synapsin-like immunoreactivity (SYNir; red) and the nuclear marker (blue).** Abbreviations: AcN acessory lobe, AMPN anterior medial, protocerebral neuropil, AnN antenna 2 neuropil, CA cerebral artery, CB central body, LAN lateral antenna 1 neuropil, mF median foramen, OGT olfactory globular tract, ON olfactory lobe, PMPN posterior medial protocerebral neuropil, PT protocerebral tract, number 9 identifies cell cluster (9).

**Figure 2:**
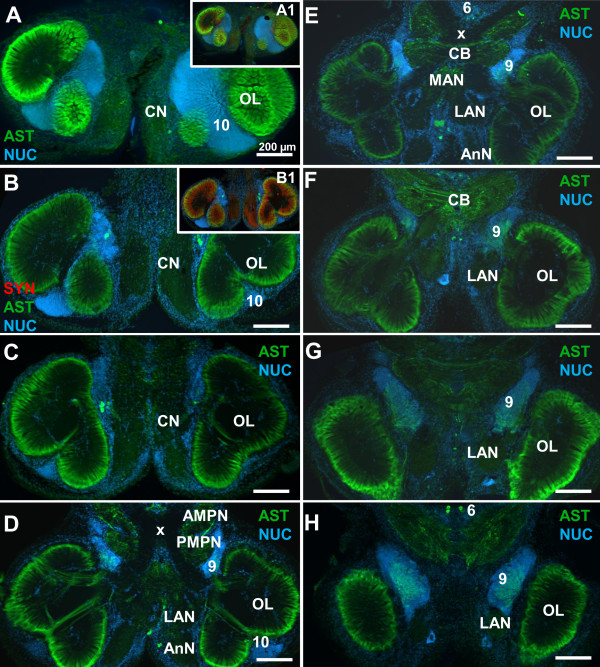
**A- type allatostatin-like immunoreactivity (ASTir) in the brain of *C. clypeatus*, low power views (conventional fluorescence with Apotome structured illumination) of a dorsal (A) to ventral (H) series of sections double labeled for ASTir (green) and the nuclear marker (blue).** Insets **A1** and **B1** show additional labelling for synapsin (red). Abbreviations: Numbers 6, 9, 10 identify cell clusters, AMPN anterior medial protocerebral neuropil, AnN antenna 2 neuropil, CB central body, CN columnar neuropil, LAN lateral antenna 1 neuropil, OL olfactory lobe, PMPN posterior median protocerebral neuropil, x chiasm of the olfactory globular tract. The scale bar is 200 μm in all images.

The general layout of the brain in *C. clypeatus* based on synapsin labelling was analysed in detail by Harzsch and Hansson [18] and therefore will be only briefly reviewed here (Figure 1). In many respects it closely conforms to that of other decapod malacostracan crustaceans [8,9,47-49]. It is composed of a protocerebrum that is divided into a lateral part housed in the eyestalk and associated with the compound eyes and a median part that houses the central complex. The deutocerebrum receives afferents from the first pair of antennae, the mechanosensory input of which is processed in the lateral and median antennae 1 neuropils. The chemosensory input from the same appendages is processed in the olfactory lobes, which are closely associated with the accessory lobe. The tritocerebrum is associated with the second pair of antennae that provide input to the antenna 2 neuropil. The immunolocalization of A-type allatostatins (ASTs; green in Figure 1) combined with a general marker for synaptic neuropil (red in Figure 1) and nuclear counter stain (blue in Figure 1) reveals a wide distribution of this peptide throughout the median brain of *Coenobita clypeatus,* especially in the central complex of the protocerebrum and the olfactory lobes, but also the eyestalk neuropils (see below). A dorsal to ventral section series featuring A-type allatostatin immunoreactivity (ASTir; green) and the nuclear (blue) counter stain (Figure 2) provides evidence for intense labelling for the neuropeptide in the synaptic compartments of the olfactory lobe, the olfactory glomeruli. Most ASTir somata that innervate the glomeruli are located in cell cluster (9). The ASTir somata that innervate the protocerebrum are located in cell cluster (6). The nomeclature of cell clusters with numbers in brackets is according to that suggested by Sandeman and co-workers [47,48]. 

### A-type allatostatin-like immunoreactivity (ASTir) in the protocerebrum

**Figure 3:**
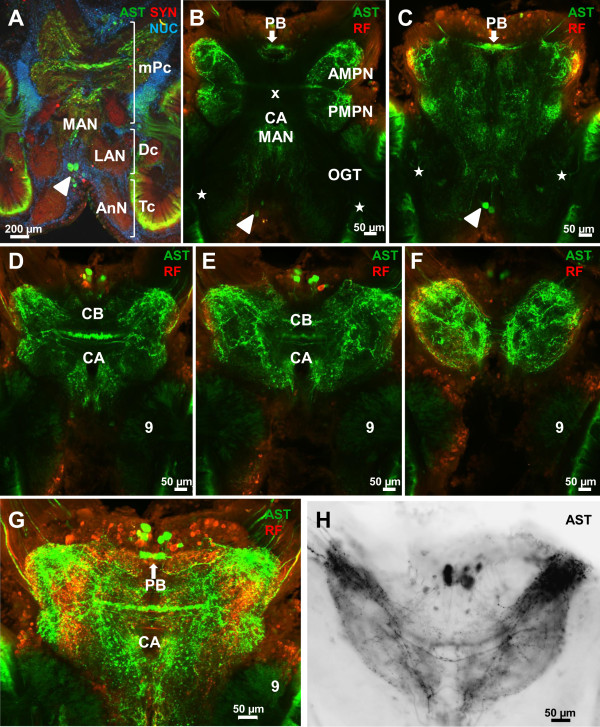
**Details of the median protocerebrum (mPc), the deutocerebrum (Dc), and the tritocerebrum (Tc), conventional fluorescence with Apotome structured illumination (A), and confocal laser scanning microscopy (B-H). A** low power overview triple labeled for ASTir (green), synapsin (SYN; red) and the nuclear marker (blue). **B**-**F**: a dorsal (**B**) to ventral (**F**) series of confocal optical sections showing ASTir (black-white inverted images). **G**, **H** higher magnifications of the median protocerebrum with central complex. G shows a double labeling against ASTir and RFir. **H** shows the allatostatin channel only (image is black/white inverted). The arrowheads indicate single ASTir neurons, asterisks point the processes of allatostatin immunoreactive local interneurons. Abbreviations: 9 cell cluster (9), AMPN anterior medial protocerebral neuropil, AnN antenna II neuropil, CA cerebral artery, CB central body, LAN lateral antenna I neuropil, MAN median antenna I neuropil, PB protocerebral bridge, OGT olfactory globular tract, PMPN posterior median protocerebral neuropil, x chiasm of the olfactory globular tract.

The protocerebrum is filled with a loose network of ASTir fibers that shows the typical subdivision of the protocerebral neuropil into an anterior and a posterior component, the anterior (AMPN) and posterior medial protocerebral neuropils (PMPN; Figure 3A-D). This subdivision is most obvious at the level of the central body (see below), whereas more ventrally and more dorsally such a distinction is not possible. The source of this labeling includes fibres associated with the protocerebral tract (PT; Figure 3G) and ca. 20 ASTir somata in cluster (6) that extend their axons into the protocerebral neuropils and the central body (Figures 1, 2H, 3B-F). The central body (CB) is an unpaired neuropil extending across the midline, embedded between the anterior and posterior medial protocerebral neuropils and displaying strong ASTir across its entire width (Figure 3). A thick ASTir commissural fiber bundle accompanies the central body posteriorly (Figure 1, 3E, H). Behind these commissural fibers, the cerebral artery (CA) pierces the brain in a dorso-ventral direction (Figures 1, 3D, E). The animal’s protocerebral bridge (PB) also displays strong ASTir and is associated with distinct immunolabelled fibres in the protocerebral tract (Figure 3B, C). Slightly dorsal to the central body lies the chiasm of the olfactory globular tract (x in Figures 2D, E, 3B). This tract does not display any ASTir but is visible as a negative imprint (Figure 3B) in the otherwise labeled protocerebral neuropils. This tract originates in the olfactory lobes and carries the axons of olfactory projection neurons (cluster 10) to the lateral protocerebrum (see Harzsch and Hansson for details [18]). In the protocerebral tract that links the anterior median protocerebral neuropil to the lateral protocerebrum, numerous ASTir fibers are present (Figure 3B-G). Double labeling reveals the presence of many neurons with RFamide-like immunorectivity (RFir) in cell cluster (6) but not any co-labelling with ASTir in neuronal somata. However, both ASTir and RFir are closely associated in the protocerebral neuropils and most likely some co-labelling is present (Figure 3G). 

### ASTir in the deutocerebrum: the olfactory lobes

**Figure 4:**
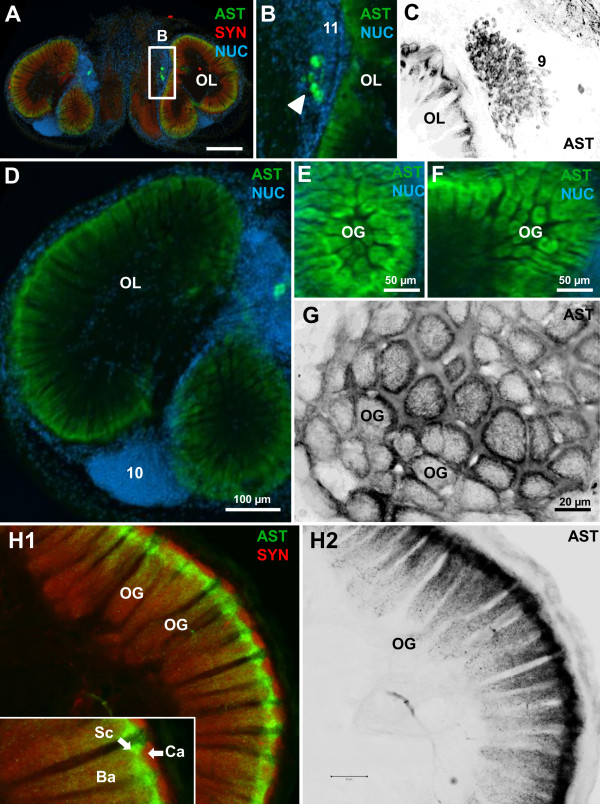
**Details of ASTir in the olfactory lobes (OL). A, B, D-F show conventional fluorescence with Apotome structured illumination, C, G, H are images obtained by confocal laser scanning microscopy. A** overview of a section triple labeled for ASTir (green), synapsin (red) and the nuclear marker (blue). The boxed area in **A** is shown in a higher magnification on **B** and displays a group of strongly immunoreactive somata of neurons within the cell cluster (9). **C** higher magnification of ASTir local olfactory interneurons in cluster (9). **D**, **E**, **F**: higher magnification of tangential section of olfactory lobes in different perspective, double labeled for allatostatin (green) and nuclear counter stain (blue), showing strong ASTir in all olfactory glomeruli. **G**: a higher magnification of the olfactory glomeruli (OG) shows that the ASTir is strongly concentrated in the periphery of the glomeruli whereas medium staining intensity is visible in the central core. **H1**, **H2**, and inset: longitudinal sections of the olfactory glomeruli show that ASTir is concentrated in the subcap (Sc) and base (Ba) region of the glomeruli. The subcap of neighboring glomeruli is connected by ASTir fibres. **H1** and the higher magnification in the inset display double staining for A-type allatostatin (green) and synapsin (red), **H2** shows the allatostatin channel only (image is black/white inverted). Abbreviations: 10 cell cluster (10), Ca cap, Ba Base, OG olfactory glomeruli, OL olfactory lobe, Sc subcap.

**Figure 5:**
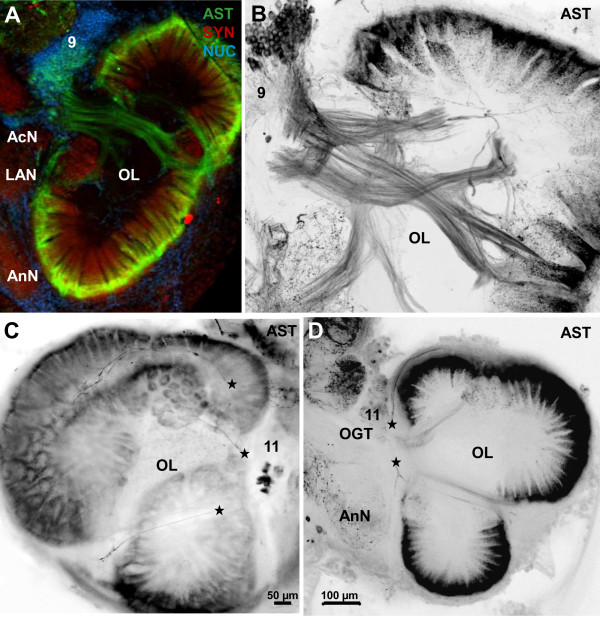
**Details of ASTir in the olfactory lobes (OL) as seen in conventional fluorescence with Apotome structured illumination (A) as well as confocal laser scanning microscopy (B-D). A **shows the olfactory lobe triple immunolabeled for ASTir (green), synapsin (red) and the nuclear marker (blue). **B**-**D** the allatostatin channel only (images are black/white inverted), showing the distribution of ASTir local interneurons and their processes. ASTir fibers from local interneurons in cluster (11) penetrate into the olfactory lobes and divide into thinner which most likely target the peripheral layer of the olfactory glomeruli from the outside. Abbreviations: 11 cell cluster (11) of local interneurons, AcN accessory neuropil (lobe), AnN antenna 2 neuropil, LAN lateral antenna 1 neuropil, OGT olfactory globular tract, OL olfactory lobe.

**Figure 6:**
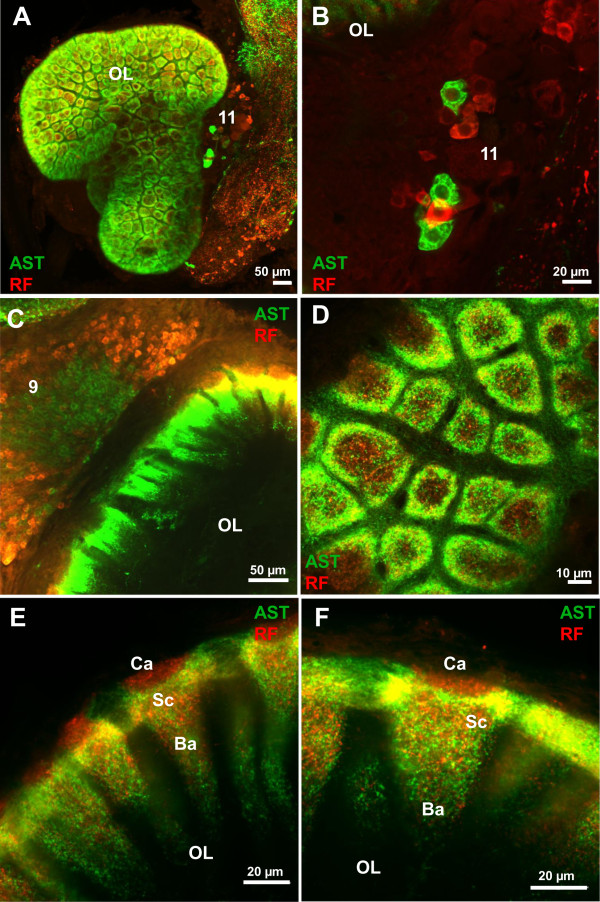
**Details of ASTir (green) and RFamide-like immunoreactivity (red) in the olfactory lobes (OL) as seen in confocal laser scanning microscopy. A** the olfactory lobe in a projection of a series of confocal sections. **B** and **C** show two different types of local interneurons (note the spatial separation of AST and RFir somata in cluster 9 on C). **D** transverse and **E**, **F** longitudinal views of the glomeruli; for details see text. Abbreviations: 9, 11 cell cluster (9) and (11) of local interneurons, Ba base, Ca cap and Sc subcup of glomerulus, OL olfactory lobe.

The bipartite olfactory lobes (OL; see Figures 2, 4A, D) dominate the brain of *C. clypeatus*[18]. They show intense ASTir and the corresponding somata are localized in cell clusters (9) and (11) (Figure 2G, H; and arrowheads in Figure 4B), which are known to house local olfactory interneurons [8,9]. We could distinguish two morphological types of ASTir interneurons. Type one is located in cluster (9), has a soma diameter of around 10 μm and amounts to at least one hundred per side (Figures 2H, 4C, 5A,B, 6C). The axons of these interneurons penetrate into the olfactory lobe via the medial foramen (mF) in a massive bundle (Figures 1, 2D-F, 5A,B). The olfactory lobes are composed of numerous column-like units, the “olfactory glomeruli”. These are elongate, cylindrical structures, where the distal part is slightly larger than the proximal part. They are arranged in a radial array around the periphery of the bipartite olfactory lobe (Figures 2, 4D-F, H). Despite their columnar shape we will refer to these neuropil elements as glomeruli since this term is well introduced in the literature [8,9]. The afore mentioned bundle of ASTir axons splits up to target the olfactory glomeruli and gives rise to strong immunoreactivity in these. Fine neurites with strong ASTir are present throughout the entire volume of the elongate glomeruli (Figure 4A-H). 

**Figure 7:**
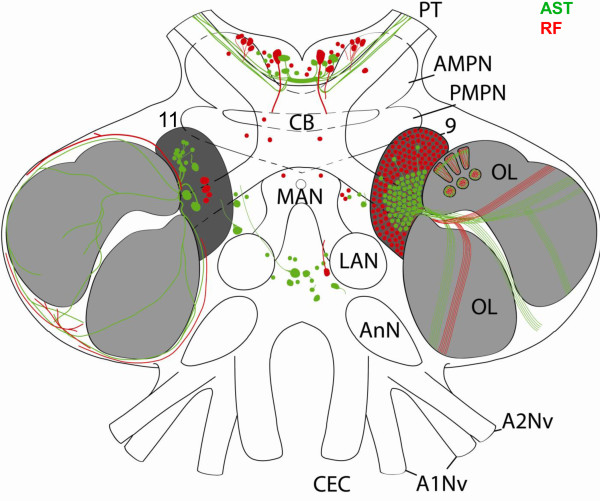
**Schematic drawing of C. clypeatus median brain, showing the distribution of A-type allatostatin (AST; green) and RFamide-like (RF; red) immunoreativity.** Two morphological types of local interneurons in cluster 9 are shown separately on each lobe. Abbreviations: 9 cell cluster (9) of local interneurons, 11 cell cluster (11) of local interneurons, A1Nv antenna 1 nerves, A2Nv antenna 2 nerve, AMPN anterior medial protocerebral neuropil, AnN antenna 2 neuropil, CB central body, OL olfactory lobe, PMPN posterior medial protocerebral neuropil, PT protocerebral tract, LAN lateral antenna 1 neuropil, MAN medial antenna 1 neuropil.

The type two of ASTir neurons, located in cluster (11) has a soma diameter of around 20 μm. The axons do not penetrate into the core of the OL as type 1 axons, but spread around the outside of the olfactory lobe in various directions (asterisks in Figure 5C,D; and schematic drawing Figure 7) to target glomeruli from the outside. Cell cluster (10), which houses the somata of the olfactory projection neurons that give rise to the olfactory globular tract [18], is devoid of any ASTir but can be visualized with the nuclear stain (Figure 2A, 4D). 

At a higher magnification, and using confocal laser-scan microscopy instead of conventional fluorescence microscopy, it becomes clear from the ASTir that the glomeruli are regionalized in two dimensions. First, looking at (optical) cross sections (Figure 4G) the label is clearly concentrated in a small rim around the periphery of the glomeruli, while the core shows a medium labelling density. Second, an analysis of the longitudinal (optical) sections of the glomeruli reveals a regionalization in cap, subcap and base regions (Figure 4H), as it is also known in other decapod crustaceans [9]. The cap region is devoid of ASTir and shows the synapsin label only (Figure 4H1; and inset), whereas the subcap is strongly immunoreactive for A-type allatostatins. Compared to the subcap, the label is weaker in the base region and shows the lowest intensity in the proximal end of the base (Figure 4H2). Horizontal sections through the olfactory lobes (Figure 5) show that once the axon bundle that emerges from the cluster (9) ASTir neurons has passed the medial foramen, it splits up into several smaller bundles (Figure 2D-F, 5A, B) that cross the coarse non-synaptic neuropil in the centre of the lobes to target the glomeruli. From our preparations it does not appear as if these ASTir axon bundles target the base of the glomeruli, but rather that the axon bundles penetrate the glomerular array in a few places to then spread out tangentially to invade the subcap regions (Figure 5A, B). This would account for the intense labeling in the subcap region as seen in longitudinal sections of the glomeruli (Figure 4H2). This tangential spreading is also visible in Figure 4G where ASTir material is visible in between the glomeruli.

### Double labeling for allatostatins and RFamide-related peptides

Harzsch and Hansson [18]. already described immunoreactivity against RFamide-related peptides (RFir) in *C. clypeatus*. With double labeling experiments we wanted to explore the spatial relationship of RFir with ASTir in the olfactory system and found a close association of these two neuropeptide systems (Figure 6A). Large RFir somata are located in immediate vicinity to the type two ASTir somata (Figure 6B) in cluster (11). Furthermore, around one hundred RFir somata are located in cluster (9) close to the ASTir type 1 neurons. Interestingly, there is a clear spatial separation with the ASTir neurons arranged medially within cluster (9) and RFir neurons surrounding these (Figure 7). This spatial separation persists within the glomeruli. Whereas in longitudinal (optical) sections it appears that ASTir and RFir are co-expressed in much of the subcap and base (Figure 6E, F), transverse (optical sections) show that ASTir is concentrated in the outer ring of the subcap (see above and Figure 4G), whereas RFir material is concentrated more medially in the core (Figure 6D). Furthermore, in the longitudinal (optical) sections it becomes clear that that RFir material is also localized in a narrow region at the interface of cap and core but that this interface region is devoid of ASTir (Figure 6E, F). 

### Other neuropils in the deutocerebrum and the tritocerebrum

The deutocerebrum also comprises the median antenna 1 neuropil (MAN) that extends across the brain posterior to the protocerebrum behind the cerebral artery. The MAN is on both sides flanked by the arms of the olfactory globular tract. Together with the lateral antenna 1 neuropils (LAN; see Figure 1) it is devoted to processing mechanosensory input from the first pair of antennae. The MAN and LAN display a moderate level of ASTir that originates from five to ten posteriorly located somata (arrowheads in 3A, B). We did not detect any ASTir in the accessory lobe (AcN; see Figure 1), which is a conspicuous neuropil of the *C. clypeatus* brain and is composed of about 50–80 small, spherical glomeruli (see Harzsch and Hansson for details [18]).

The main structure of the tritocerebrum in the *C. clypeatus* brain is the antenna 2 neuropil (AnN) which receives mechanosensory afferents from the second pair of antennae and contains the motoneurons that control the movements of the second antennae [47,50]. This neuropil is filled with a network of weakly AST-immunolabelled fibres (Figure 2D).

### Secondary olfactory centers in the eyestalk: the lateral protocerebrum - medulla terminalis and hemiellipsoid body

**Figure 8:**
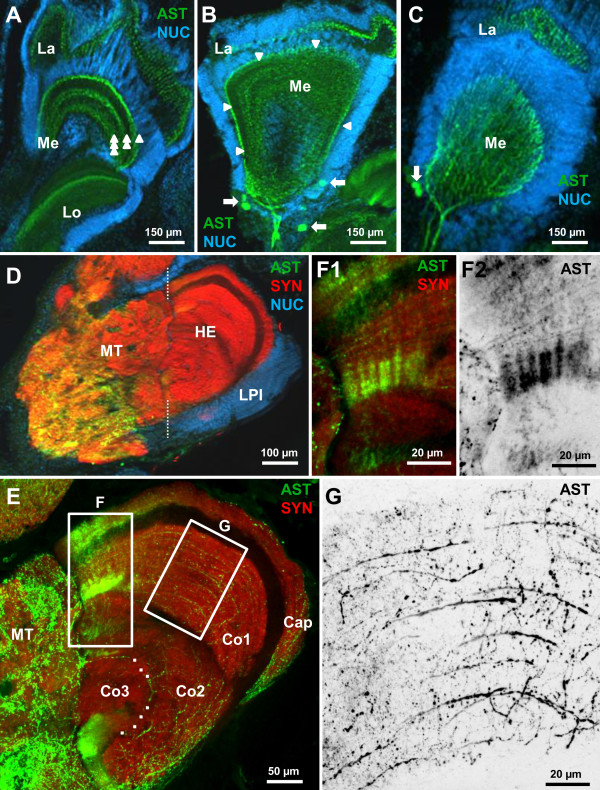
**Details of ASTir in the eyestalk neuropils AST immunoractivity.A**,**B**,**C**: higher magnifications (conventional fluorescence with Apotome structured illumination), double labelling for ASTir (green) and the nuclear marker (blue). ASTir material in the medulla is arranged in three parallel layers, labeled by single, double, and triple arrowheads in A. Arrowheads in B indicate the outer (distal) layer of ASTir neurites of the medulla. Arrows in B and C identify ASTir somata. **D** higher magnification of the lateral protocerebrum (triple labelling, conventional fluorescence with Apotome structured illumination). **E** confocal-laser scan microscopic images (double labelling) of the hemiellipsoid body to show its subdivisions. Dashed line indicates the border of medulla terminalis and hemiellipsoid body. The boxed areas are magnified in **F** and **G**.

The medial protocerebrum is connected to the eyestalk neuropils *via* the protocerebral tract (PT; Figure 1). The eyestalks house a substantial part of the brain including the lateral protocerebrum, which is composed of the medulla terminalis (MT) and the hemiellipsoid body (HE), two neuropils that in addition to receiving visual input are also targeted by the axons of olfactory projection neurons. Furthermore, the four optic neuropils lamina (La), medulla (Me), lobula (Lo) and a small proximal lobula neuropil (LoP) are enclosed in the eyestalks (Figure 8,; and compare Harzsch and Hansson [18]). Because we could not determine the dorso-ventral orientation of dissected neuropils we found it impossible to generate sections that are unambiguously oriented e.g. in the horizontal plane. 

An analysis of the lateral protocerebrum showed strong immunolabeling of rather coarse neurites throughout the entire medulla terminalis (Figure 8D). Because of our difficulties to obtain a reliable orientation of the sections, we could not recognize any conspicuous substructures within the medulla terminalis. The hemiellipsoid body is a large spherical neuropil (ca. 300 μm in diameter; compare [18,44]), that is associated with a compact, laterally situated cluster of densely packed neurons, the lateral protocerebral interneurons (LPI; Figure 8D). The hemiellipsoid body can be easily subdivided into the peripheral cap neuropil (Cap), and the core neuropils I-III (Co1-Co3; compare [44]) separated by an unlabelled first and second intermediate layer (IL1 and IL2, respectively) (Figure 8E) that are made up of neurite bundles from the lateral protocerebral interneurons (“globuli cells”; see [44]). Cap, core I and core II neuropils all display ASTir that appear homogenously distributed in low power views, where single fibres were not recognizable (data not shown). The source of this innervation with neurites that contain A-type allatostatins could not be determined. The cluster of lateral protocerebral interneurons that is associated with the hemiellipsoid body did not contain any ASTir somata. The core III neuropil was devoid of any ASTir. Higher magnifications of the hemiellipsoid body with confocal laser-scan technique provided a clearer picture of the immunoreactive fibers in Cap, Co1 and Co2 (Figure 8F-G). Neurites with ASTir in Co1 are aligned in parallel tangential layers (Figure 8G) reminiscent of the neuropil lamellae that had previously been observed by synapsin labeling [18] and Golgi impregnations [44]. However, a different type ASTir material seems to be arranged such that it crosses the tangential fibres at a right angle (Figure 8F1, F2) suggesting a grid-like architecture of Co2 as it has been recently described based on other markers [44].

### The other eyestalk neuropils: lamina, medulla and lobula (optic neuropils)

**Figure 9:**
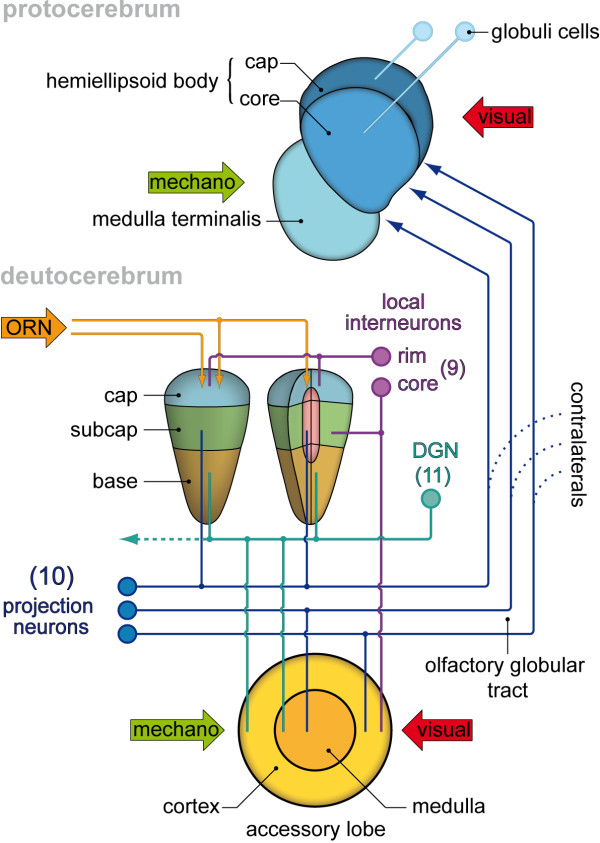
**Overview of the central olfactory pathway in a malacostracan crustacean, the crayfish (modified from [**[57]**]).** The olfactory receptor axons (orange) are the primary sensory input and innervate the cap of the olfactory glomeruli. Local interneurons (purple) in cell cluster (9) and dorsal giant neurons (serotonergic, turquoise) in cell cluster (11) are associated with the olfactory and the accessory lobe. The olfactory glomeruli are compartmentalized into the cap, subcap, and base regions (see Figure 10 for details). Local interneurons innervate specific compartments of the olfactory glomeruli and were classified as e.g. rim or core interneurons. The dorsal giant neuron (DGN) innervates both the olfactory glomeruli and the accessory lobe. The accessory lobe also shows responses to visual and mechanosensory stimuli. From the olfactory lobe and accessory lobe, processed information is relayed to the secondary computational centers in the lateral protocerebrum, the medulla terminalis and the hemiellipsoid body *via* the projection neuron tract (blue), that also provides contralateral connections. In the lateral protocerebrum, this input interacts with the intrinsic lateral protocerebral interneurons, often termed “globuli cells”. Projection neurons associated with the accessory lobe target the hemiellipsoid body whereas those asscociated with the olfactory glomeruli mostly target the medulla terminalis. The lateral protocerebrums also receives indirect mechanosensory and visual input in addition to chemosensory information and hence is a multimodal center.

In *C. clypeatus*, like in other decapod crustaceans, the visual system consists of the compound eyes and three columnar optic neuropils, lamina (La), medulla (Me) and lobula (Lo) as well as the recently discovered lobula plate. Comparing synapsin labeling, which allows for the visualization of the neuropil organization, with the distribution of ASTir reveals that the peptide is widely distributed in lamina, medulla and lobula (unclear for the lobula plate). The most distal of the three optic neuropils, the lamina, appears as a thin layer of ASTir profiles in a geometrical layout that reflects the arrangement of optic cartridges (Figure 8A-C). The origin of these peptidergic innervations could not be determined. Higher magnifications reveal that ASTir material in the medulla is arranged in three parallel layers, labeled by single, double, and triple arrowheads in Figure 8A. One layer is located at the distal margin of that neuropil (single arrowhead), the second in the centre (double arrowhead) and the third at the proximal margin of the neuropil (triple arrowhead). A tangential section through the medulla is shown in Figure 8B, where the arrowheads identify the distal layer of ASTir neurites. Thick immunolabelled fibres that reach the medulla from a proximal direction seem to be the source of this innervation (Figures. 8B, C). Furthermore, ASTir somata located close to the medulla seem to contribute to its peptidergic innervations (arrown in Figure 8B, C). The lobula is also immunoreactive for A-type allatostatins. The signal is mostly concentrated in a layer that is located in the centre of the lobula (Figure 8, 9A).

## Discussion

Considering that in the family of crustacean A-ASTs, we now know several dozens of representatives [6], their distribution in the brain of malacostracan crustaceans has received little attention. Immunolocalization experiments so far have indicated the presence of A-ASTs in the eyestalk ganglia [23,38], the central complex [23,41]) and local olfactory interneurons [17,23]. In two more comprehensive studies we have recently explored ASTir in the brains of the brachyuran crab *Carcinus maenas* and four representatives of the hermit crabs (Anomura; [39,40]). These studies have indicated a rather global distribution of A-ASTs suggesting diverse functional roles in the visual and olfactory system and will serve as a basis for comparisons with our present results which will focus on the central olfactory pathway. Because ASTir in three columnar optic neuropils (the lamina, medulla and lobula) of *C. clypeatus* is essentially similar to that of its decapods relatives *C. maenas, Pagurus bernhardus* and *Birgus latro*[39,40] and because the chemical architecture of these neuropils has been thoroughly discussed recently in crayfish [16] and also in *C. clypeatus*[18], we will not touch this aspect. The medulla terminalis will be discussed in the context of the central olfactory pathway.

### A-type allatostatins in the arthropod central olfactory pathway

**Figure 10:**
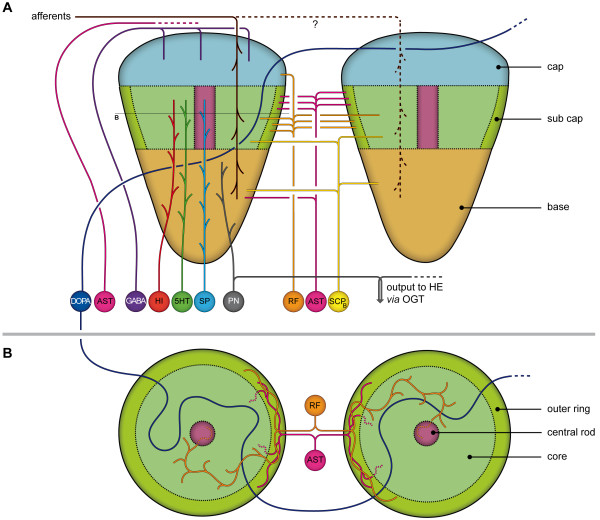
**Schematic drawing of the regionalization in decapod crustacean olfactory glomeruli and neurotransmitter complexity of local interneurons (A lateral view, B transverse view of glomeruli at the subcap level).** The scheme unites data from the spiny lobster *Panulirus argus,* various crayfish species and the hermit crab *Coenobita clypeatus* [compiled after [13,18,19,55,69,70]. The afferent axons of olfactory receptor neurons most likely have multiglomerular terminations in spiny lobsters. Abbreviations: 5HT serotonin, AST A-type allatostatin, DOPA dopamine, GABA gamma amino butyric acid, HE hemiellipsoid body neuropil, OGT olfactory globular tract, PN projection neurons, RF RFamide, SCPb small cardioactive peptide, SP substance P.

General immunolocalization studies have provided evidence that, in principle, A-type allatostatins are present in the olfactory lobes of various malacostracan crustaceans, such as the crayfish *Procambarus clarkii*[17] and *Orconectes limosus*[23], the prawn *Penaeus monodon*[51], the shore crab *Carcinus maenas* and the marine hermit crab *Pagurus bernhardus*[40], and the giant robber crab *Birgus latro*[39], a close relative of *C. clypeatus*. Here, we provide the first higher resolution analysis of this peptide`s distribution in the crustacean olfactory system. They are also present in antennal lobes of various insect species, including honeybee *Apis melifera*[46], the cockroach *Diploptera punctata*[20], the tobacco hornworm *Manduca sexta*[52], and the wingless hexapod *Lepismachilis y-signata* (Archaeognatha, [53]). In *C. clypeatus* and the crayfish [17,23] the ASTir fibres that target the olfactory glomeruli in the OL from the proximal side emerge from local olfactory interneurons in cell cluster (9). The olfactory interneurons in this cell cluster are involved in modulation of olfactory processing and, as is known mostly from studies on crayfish, clawed lobsters and spiny lobsters, synthesize a large variety of neurotransmitters (Figures 9, 10) including serotonin, histamine and GABA as well as many different neuropeptides such as RFamide related peptides, substance P, small cardiactive peptide b, orcokinins, SIFamide, and tachykinin-related peptides [10-18], review [19]. For *C. clypeatus*, so far RFamide related peptides, allatostatins and serotonin have been shown for cluster (9) neurons ( [18]; and present study). The diversity of neurotransmitter phenotypes of cluster (9) neurons is mirrored in the characteristic regionalization of malacostracan olfactory glomeruli. In longitudinal sections, cap, subcap, and base regions can be distinguished (Figure 10; and [13,14]). In cross sections of the subcap region, a concentric arrangement of three compartments is present, the central rod, core, and outer ring regions. These compartments are defined by the projection patterns of afferent olfactory sensory neurons, local olfactory interneurons, and olfactory projection neurons. For example, the central rod of the subcap is characterized by substance P expression whereas the core displays RFamide-like immunoreactivity (Figure 10). However, the fine morphology of local interneuron branchings and their synaptic connectivity as well as the whole neuronal network in the olfactory lobe of *C. clypeatus* are poorly understood and require further studies involving backfill and intracellular techniques. The present study is the first study that allowed a localization of allatostatin-like immunoreactivity from cluster (9) neurons to the outer rim region of the glomeruli (Figure 10). The second type of ASTir neurons with their large somata in cluster (11) course over the surface of the olfactory lobe and most likely penetrate into the glomeruli from the periphery. The olfactory lobes of the moth *Heliothis virescens* are also targeted by two different types of ASTir neurons one with large the other with smaller somata [54]. In the spiny lobster, Wachowiak and co-workers [55] described the physiology and anatomy of a certain type of local olfactory interneuron, the “rim neurons” (type II; see Figure 10), with large somata and neurites that travel around the periphery of the olfactory lobe to innervate the cap region of the glomeruli. The anatomy of these neurons conforms to that of the type of ASTir neurons with their large somata in cluster (11) that we describe in the present report. 

### Neurotransmitter diversity in the crustacean olfactory glomeruli

The distribution of serotonin and RFamide-like peptides in the glomeruli seems to be similar to the pattern in crayfish and spiny lobsters. Double labeling of allatostatin-like peptides with FMRFamide-like antibodies shows no co-expression of these two neuropeptides in the local olfactory interneurons, rather on the contrary: subsets of FMRFamide-like- and ASTir somata of local interneurons form distinct separate clusters that match their localized expression in the core *versus* outer rim region of the glomerular subcap (Figure 10). The available information as summarized in Figure 10 suggest that crustacean olfactory glomeruli are targeted by the axons of the olfactory receptor neurons from the outside, possibly in a multiglomerular pattern [8,9] (dashed line in Figure 10A). In addition, the neurites of the large ASTir neurons and the neurites of GABAergic neurons travel around the outside of the olfactory lobes to enter the cap of the glomeruli from the outside. This stream of incoming receptor axons and neurites of GABAergic and ASTir local interneurons from the outside meets the neurites of local interneurons with substance P, serotonin and histamine that come from the inside of the olfactory lobes to dive into the base of the glomeruli. A third class of local interneurons has neurites that target the subcap region and express RFamide-like peptides, ASTir or show immunoreactivity for small cardioactive peptide B (Figure 10). Projection neurons that target the secondary olfactory centers in the protocerebrum (the hemiellipsod bodies; see below) are the major output channel of the system. In summary, we can expect that the crustacean olfactory glomeruli house complex local circuits the connectivity within which as yet is completely unclear. Because the glomeruli of *C. clypeatus* and its close relative *B. latro* belong to the longest known for decapod crustaceans [39], and hence display a distinct pattern of regionalization, their olfactory systems form an ideal model to explore the functional relevance of glomerular compartments and diversity of local olfactory interneurons for olfactory processing in crustaceans.

### Comparison to the insect olfactory system

Although the insect studies mentioned above address different questions on the role of ASTs in the brain and thus do not always provide a detailed description of ASTir in the central olfactory pathway, there are nevertheless obvious similarities with our results in *C. clypeatus*. In insects, ASTir somata are found in a cluster housing local olfactory/antennal lobe interneurons, whose axons in honeybees invade the core of their spherical glomeruli without an evident overlap with afferent projections [46]. In general, regionalization of the mostly spherical insect olfactory glomeruli is much less pronounced than that in crustaceans, suggesting that the latter may have more elaborate local computing capacities within their glomeruli. What is more, the number of glomeruli in malacostracans also seems to be considerably higher than that in insects, amounting to well over thousand in several malacostracan species [56,57]. Based on the pattern of ASTir and our general knowledge on the architecture of all other involved neuronal players, the cap of bee glomeruli may be equivalent to the cap in decapods crustacean glomeruli, and the bee core may be equivalent to the crustacean base region. In bee glomeruli, Kreissl et al. [46] reported a distinct border region between cap and core, where ASTir profiles are concentrated. Based on our analysis of ASTir in the *C. clypeatus* glomeruli, we suggest that the outer ring of the subcap region of malacostracan glomeruli corresponds to this border region in the bee.

Neither in Malacostraca nor in insects, ASTir has been observed in the somata of projection neurons, which means that ASTs are not responsible for transferring olfactory information to higher brain centers (mushroom bodies in case of insects, hemiellipsoid body and terminal medulla in crustaceans), but rather are involved in a modulation of the olfactory signal at the level of the deutocerebrum. Though ASTs are documented to exert an inhibitory myomodulatory function [34,36], their precise role in olfaction is so far unclear and AST-like peptide receptors in crustaceans is yet to be characterized. In the honeybee, a subset of local interneurons coexpress ASTs with GABA, which could suggest that ASTs also act as inhibitory neuromodulators and help to sharpen the olfactory signal [46].

### Second-order olfactory neuropils: the crustacean hemiellipsoid bodies *versus* the insect mushroom bodies

In malacostracan crustaceans, the axons of olfactory projection neurons relay olfactory information from first-order processing areas, the olfactory lobes, to second-order olfactory neuropils, the hemiellipsoid bodies in the protocerebrum (Figure 9; [8,9,49]). We already noted previously that both first- and second-order olfactory neuropils in *C. clypeatus* are substantially enlarged in comparison to marine decapods crustaceans [18], an observation that was later verified in *Coenobita’s* larger relative, the giant robber crab *Birgus latro*[39]. In *C. clypeatus*, the estimated number of projection neurons is 20,000 per side [44], and ca. 160,000 in *Birgus latro*[39]. In the hemiellipsod bodies, the projection neuron input interacts with intrinsic interneurons in a rectilinear array [44], ca. 125,000 neurons per side in *C. clypeatus* and ca. 250,000 in *B. latro*[39]. Both species devote an enormous amount of neuronal tissue to processing chemosensory input. In a recent comparative study [44] we showed that the neuronal networks in the *C. clypeatus* hemiellipsoid bodies share more similarities with that in insect mushroom bodies, also second-order olfactory neuropils, than we had previously expected. Comparisons of the morphology, ultrastructure, and immunoreactivity of the hemiellipsoid body of *C. clypeatus* and the mushroom body of the cockroach *P. americana* revealed both a layered motif provided by rectilinear arrangements of extrinsic and intrinsic neurons as well as a microglomerular organization. These findings suggest that the central olfactory pathways of malacostracan crustaceans (Figure 9) and insects share an ancestral computational circuit: in the olfactory glomeruli in the deutocerebrum, afferents from olfactory sensory neurons from antenna one interact with various local olfactory interneurons and dendrites of olfactory projection neurons. The axons of the latter project to protocerebral areas where they interact with neurites of intrinsic cells (called Kenyon cells in insects) in a rectilinear array. A convergent multiplication of this basic circuit during evolution of the divergent insect and malacostracan lineages to increase computational power has generated different morphological phenotypes of the olfactory centers that have masked the deep homology of their basic neuronal networks.

## Material and methods

### Animals

Adult specimens of *Coenobita clypeatus* (Herbst, 1791; Anomura, Coenobitidae) were obtained from the “Zoologischer Großhandel Peter Hoch” (August Jeanmaire Str. 12, 79183 Waldkirch, Germany; http://www.hoch-rep.com/). The animals (ca. 5–8 cm total length) were anaesthetized for at least one hour on ice and then their brains were dissected in phosphate buffered saline (0.1M PBS, pH 7.4). Killing of the animals was carried out in accordance with the national ethical guidelines (“genehmigungsfreien Versuchsvorhabens nach § 8a Abs. 1 und 2 des Tierschutzgesetzes Deutschland vom 18. Mai 2006 BGBl. I S. 1206”) including notification and consent of the responsible administrative authorities of the University of Greifswald.

### Immunochemistry

The isolated brains and eyestalks were fixed overnight in 4% PFA in 0.1M PBS, pH 7.4 at 4°C. After fixation the tissues were washed for 4 hours in several changes of PBS and subsequently processed as whole mount after carefully removing the neurolemma using forceps or sectioned (80 μm) with a HM 650 V Vibrating Blade Microtome (Microm). Overnight permeabilization in PBTx (0.3% Tx-100 in 0.1 M PBS, pH 7.4) at 4°C of the specimens was followed by an incubation in 1% NGS in PBS TX for at least 2 hours and an incubation in the primary antibodies overnight at 4°C (sections) or for 2 days at 4°C (whole mounts). We used the following reagents for the immonohistolabelling labeling experiments: polyclonal rabbit or monoclonal mouse anti-type A allatostatin (dilution 1:1000 [45])*,* in some experiments combined with monoclonal mouse anti-synapsin “SYNORF1“ antibody (dilution 1:20 [58] or rabbit anti-FMRFamide antiserum (Immunostar Inc., dilution 1:100). Subsequently, all tissues were washed in several changes of PBS for 2 hours at room temperature and incubated in a mix of the secondary anti-rabbit Alexa Fluor 488 antibodies (Invitrogen, Molecular Probes), and secondary anti-mouse Cy3 antibodies (Jackson ImmunoResearch Laboratories Inc.) and the nuclear dye bisbenzimide as a histochemical counterstain (0.05%, Hoechst H 33258) for another 4 hours. Finally, the tissues were washed for at least 2 hours in several changes of PBS at room temperature and mounted in Mowiol (sections) or dehydrated in an ethanol series (50, 70, 90, 2 x 100%, 10 min each), and mounted in methylsalicilate (whole mounts). The specimens were viewed with a Zeiss AxioImager equipped with the Zeiss Apotome structured illumination device for optical sectioning (“grid projection”; http://www.zeiss.de/apotome). Digital images were processed with the Zeiss AxioVision software package. In addition, specimens were analyzed with the laser scanning microscope Zeiss LSM 510 Meta. Double-labeled specimens were generally analyzed in the multi-track mode in which the two lasers operate sequentially, and narrow band-pass filters were used to assure a clean separation of the labels and to avoid any crosstalk between the channels. All images were processed in Adobe Photoshop using global picture enhancement features (brightness/contrast).

### Specificity of the antisera

#### Allatostatin

We used an antiserum that was raised against the *Diploptera punctata* (Pacific beetle cockroach) A-type Dip-allatostatin I, APSGAQRLYGFGLamide, coupled to bovine thyroglobulin using glutaraldehyde [45] and that previously has been used to localize A-ASTs in crustacean and insect nervous systems [23,32,41,45,46]. Competitive ELISA with DIP-allatostatin I, II, III, IV and B2 showed that the antiserum is two orders of magnitude more sensitive to Dip-allatostatin I than to Dip-allatostatins II, III, IV, and B2 [45]. Vitzthum and coauthors [45] have reported that the antiserum displays no cross-reactivity with corazonin, CCAP, FMRFamide, leucomyosuppression, locustatachykinin 11, perisulfakinin, and proctolin as tested by non-competitive ELISA. Preadsorption of the diluted antisera against Dip-allatostatin I, GMAP and *Manduca sexta* allatotropin with 10 μM of their respective antigens abolished all immunostaining in brain sections of *Schistocerca gregaria*[45]. A sensitive competitive enzyme immunoassay (EIA) confirmed the high specificity of the antiserum for A-type Dip-allatostatin I [17]. In the brains of the honey bee *Apis mellifera*, preadsorption controls with AST I and AST VI completely abolished all staining of the antiserum [46]. Preadsorption of the antiserum with AST-3 was reported to abolish all labelling in the stomatogastric nervous system of the crab *Cancer pagurus*, the lobster *Homarus americanus* and the crayfish *Cherax destructor* and *Procambarus clarki*[32]. We also performed a preadsorption test and preincubated the antiserum with 200 μg/ml A-type Allatostatin I (Sigma, A9929; 16 h at 4°C) which abolished all staining in *C. clypeatus* brains. Kreissl and coworkers [46] suggested that in the honeybee, this particular antiserum binds to A-AST isoforms that share a –YSFGLamide core. Most A-ASTs in decapods crustaceans display the C-terminal motifs -YAFGLamide, -YGFGLamide, -YNFGLamide, or -YSFGLamide [17,22,23] so that it seems reasonable to conclude that, as in insects, the antiserum we used recognizes all crustacean A-ASTs that share a -Y*X*FGLamide core.

Therefore, we will refer to the labeled structures in our specimens as "Allatostatin A-like immunoreactivity (ASTir)" throughout this manuscript.

### FMRF-amide

The tetrapeptide FMRFamide and FMRFamide-related peptides (FaRPs) are prevalent among invertebrates and vertebrates and form a large neuropeptide family with more than 50 members all of which share the RFamide motif [1,59-61]. In malacostracan Crustacea, at least twelve FaRPs have been identified and sequenced from crabs, shrimps, lobsters, and crayfish [26,61], which range from seven to twelve amino acids in length and most of them share the carboxy-terminal sequence Leu-Arg-Phe-amide. The utilized antiserum was generated in rabbit against synthetic FMRFamide (Phe-Met-Arg-Phe-amide) conjugated to bovine thyroglobulin (DiaSorin; Cat. No. 20091; Lot No. 923602). According to the manufacturer, immunhistochemistry with this antiserum is completely eliminated by pretreatment of the diluted antibody with 100 μg/ml of FMRFamide. We repeated this experiment and preincubated the antiserum with 100 μg/ml FMRFamide (Sigma; 16 h, 4°C) resulting in a complete abolishment of all staining. In another approach we omitted the primary antibody which also abolished all staining. Because the crustacean FaRPs known so far all share the carboxy-terminal sequence LRFamide, we conclude that the DiaSorin antiserum that we used most likely labels any peptide terminating with the sequence RFamide. Therefore, we will refer to the labeled structures in our specimens as “RFamide-like immunoreactivity” (RFir) throughout the paper.

### Synapsin

The monoclonal mouse anti-*Drosophila* synapsin “SYNORF1” antibody (kindly provided by E. Buchner, Universität Würzburg, Germany) was raised against a *Drosophila* GST-synapsin fusion protein and recognizes at least four synapsin isoforms (ca. 70, 74, 80, and 143 kDa) in western blots of *Drosophila* head homogenates [58]. In Western blot analysis of crayfish homogenates, this antibody stains a single band at ca. 75 kDa [62]. We have conducted a western blot analysis comparing brain tissue of *Drosophila* and *Coenobita clypeatus*[18] in which the antibody provided identical results for both species staining one strong band around 80–90 kDa and a second weaker band slightly above 148 kDa. This previous analysis strongly suggested that the epitope which SYNORF 1 recognizes is strongly conserved between the fruit fly and the hermit crab. Similar to *Drosophila*, the antibody consistently labels brain structures in representatives of all major subgroups of the malacostracan crustaceans [39,40,56,63-67] in a pattern that is consistent with the assumption that this antibody does in fact label synaptic neuropil in Crustacea. The antibody also labels neuromuscular synapses both in *Drosophila* and in Crustacea [64]. These close parallels in the labeling pattern of SYNORF1 between *Drosophila* and various Crustacea strengthens the claim that it also recognizes crustacean synapsin homologs. According to the preceded terminology, we will refer to the labelled structures as “synapsin-like immunoreactivity (SYNir)” throughout this manuscript.

### Nomenclature

The neuroanatomical nomenclature, used in this manuscript is based on that proposed by Sandeman and co-workers [47,48] with some modifications adopted from Harzsch and Hansson [18]. Cell clusters are numbered in brackets following the syntax proposed by Sandeman and co-workers [47,48]. All other aspects of the basic neuroanatomical nomenclature are according to the glossary by Richter et al. [68].

## Abbreviations

A1Nv: Antenna 1 nerves; A2Nv: Antenna 2 nerve; AcN: Acessory lobe; AMPN: Anterior medial protocerebral neuropil; AnN: Antenna 2 neuropil; AST: A-type allatostatin; ASTir: A-type allatostatin-like immunoreactivity; Ba: Base; CA: Cerebral artery; Ca: Cap; CB: Central body; CN: Columnar neuropil; Co1: Core neuropil 1; Co2: Core neuropil 2; Co3: Core 3 neuropil; DOPA: Dopamine; GABA: Gamma amino butyric acid; HE: Hemiellipsoid body; La: Lamina; LAN: Lateral antenna 1 neuropil; Lo: Lobula; LPI: Lateral protocerebral interneurons; MAN: Median antenna I neuropil; Me: Medulla; mF: Median foramen; mPc: Median protocerebrum; MT: Medulla terminalis; OGT: Olfactory globular tract; OG: Olfactory glomeruli; OL: Olfactory lobes; ON: Olfactory lobe; SYNir: Synapsin-like immunoreactivity; PB: Protocerebral bridge; PMPN: Posterior medial protocerebral neuropil; PN: Projection neurons; PT: Protocerebral tract; RF: RFamide; Sc: Subcap; SCPb: Small cardioactive peptide; SP: Substance P; Tc: Tritocerebrum; X: Chiasm of the olfactory globular tract; 5HT: Serotonin; Numbers: 6 9, 10, 11 identifies cell cluster.

## Competing interests

The authors declare that they have no competing interests.

## Authors’ contributions

MP carried out part of the immunohistochemical experiments and microscopic analysis and assisted in drafting the manuscript. OT carried out part of the immunohistochemical experiments and microscopic analysis and assisted in drafting the manuscript. HA participated in the design of the study and assisted with the immunohistochemical experiments. BSH participated in the design of the study and helped to draft the manuscript. SH conceived and coordinated the study and drafted the manuscript. All authors read and approved the manuscript.
